# Expression and Processing of a Small Nucleolar RNA from the Epstein-Barr Virus Genome

**DOI:** 10.1371/journal.ppat.1000547

**Published:** 2009-08-14

**Authors:** Roland Hutzinger, Regina Feederle, Jan Mrazek, Natalia Schiefermeier, Piotr J. Balwierz, Mihaela Zavolan, Norbert Polacek, Henri-Jacques Delecluse, Alexander Hüttenhofer

**Affiliations:** 1 Innsbruck Biocenter, Division of Genomics and RNomics, Innsbruck Medical University, Innsbruck, Austria; 2 German Cancer Research Center, Department of Virus-Associated Tumours, Heidelberg, Germany; 3 Department of Biological Chemistry, David Geffen School of Medicine, University of California Los Angeles, Los Angeles, California, United States of America; 4 Innsbruck Biocenter, Division of Cell Biology, Innsbruck Medical University, Innsbruck, Austria; 5 Biozentrum, Swiss Institute of Bioinformatics, University of Basel, Basel, Switzerland; Duke University Medical Center, United States of America

## Abstract

Small nucleolar RNAs (snoRNAs) are localized within the nucleolus, a sub-nuclear compartment, in which they guide ribosomal or spliceosomal RNA modifications, respectively. Up until now, snoRNAs have only been identified in eukaryal and archaeal genomes, but are notably absent in bacteria. By screening B lymphocytes for expression of non-coding RNAs (ncRNAs) induced by the Epstein-Barr virus (EBV), we here report, for the first time, the identification of a snoRNA gene within a viral genome, designated as v-snoRNA1. This genetic element displays all hallmark sequence motifs of a canonical C/D box snoRNA, namely C/C′- as well as D/D′-boxes. The nucleolar localization of v-snoRNA1 was verified by *in situ* hybridisation of EBV-infected cells. We also confirmed binding of the three canonical snoRNA proteins, fibrillarin, Nop56 and Nop58, to v-snoRNA1. The C-box motif of v-snoRNA1 was shown to be crucial for the stability of the viral snoRNA; its selective deletion in the viral genome led to a complete down-regulation of v-snoRNA1 expression levels within EBV-infected B cells. We further provide evidence that v-snoRNA1 might serve as a miRNA-like precursor, which is processed into 24 nt sized RNA species, designated as v-snoRNA1^24pp^. A potential target site of v-snoRNA1^24pp^ was identified within the 3′-UTR of BALF5 mRNA which encodes the viral DNA polymerase. V-snoRNA1 was found to be expressed in all investigated EBV-positive cell lines, including lymphoblastoid cell lines (LCL). Interestingly, induction of the lytic cycle markedly up-regulated expression levels of v-snoRNA1 up to 30-fold. By a computational approach, we identified a v-snoRNA1 homolog in the rhesus lymphocryptovirus genome. This evolutionary conservation suggests an important role of v-snoRNA1 during γ-herpesvirus infection.

## Author Summary

Epstein-Barr virus (EBV) infects about 90% of people worldwide and is associated with different types of cancer. So far, only two large virus-encoded non-coding RNAs (EBER1 and EBER2) and 25 microRNAs (miRNAs) have been identified in the EBV genome. In this study, we report identification of the first member of another abundant non-coding RNA class, a small nucleolar RNA (snoRNA), designated as v-snoRNA1. We show that v-snoRNA1 is located in the nucleolus and interacts with the same proteins as reported for canonical eukaryal snoRNAs. Its biological function is consistent with its high conservation in a distantly related simian herpesvirus genome. Interestingly, v-snoRNA1 might serve as a miRNA-like precursor, which is processed into a 24 nt sized RNA species, designated as v-snoRNA1^24pp^. The viral DNA polymerase BALF5 was identified as a potential target for v-snoRNA1^24pp^. Taken together, these experiments strengthen the crucial function of v-snoRNA1 in EBV infection.

## Introduction

The Epstein-Barr virus (EBV), a member of the γ-herpesvirus subfamily, possesses a large (170 to 180 kb) double-stranded DNA genome. EBV infection is etiologically linked with various cancers of the lymphoid and epithelial lineages that include Burkitt's lymphoma (BL), Hodgkin's disease, nasopharyngeal carcinoma (NPC) and post-transplant lymphoproliferate disease (PTLD) [1]–[4]. *In vitro* and *in vivo*, EBV transforms normal B cells through establishment of a type III latency during which a restricted set of viral genes is expressed (eight Epstein-Barr nuclear antigens and two latent membrane proteins) [5]. More restricted expression patterns such as latency type II in NPC and latency type I in BL have also been characterized. In fact, recent work on Burkitt's lymphoma has shown that a subset of these tumours display a latency pattern intermediate between latency I and III showing that the boundaries between the latency types are not always sharply established as initially thought [6].

More then two decades ago, the group of J. Steitz discovered two highly abundant ∼170-nt long non-coding RNAs (ncRNAs) in the EBV genome, designated as Epstein-Barr encoded RNAs (EBER1 and EBER2) [7]. EBER RNAs have subsequently been shown to bind to human ribosomal protein L22. However, no unequivocal biological functions could be assigned to EBER transcripts, up till now [8]. The list of non-coding RNAs encoded by EBV has since rapidly expanded with the recent discovery of 25 microRNAs (miRNAs) [9]–[14].

In addition to miRNAs, numerous other ncRNAs have been discovered in all three domains of life, i.e. Archaea, Bacteria and Eukarya, as well as in various viruses [15],[16]. A large number of these ncRNA species were found to be involved in multiple regulatory functions including cellular differentiation and development, chromatin architecture, transcription and translation, alternative splicing, RNA editing, virulence and stress responses [17]–[20].

Small nucleolar RNAs (snoRNAs) consist of more than 200 stable ncRNA species in Eukarya of about 60 to 300 nt in size which are located in a sub-nuclear compartment, the nucleolus [21],[22]. SnoRNAs guide nucleotide modifications within ribosomal RNAs (rRNAs) or spliceosomal RNAs (snRNAs), i.e. 2′-O-ribose methylation or pseudouridylation, respectively. The snoRNA class has been identified in Archaea and Eukarya, but not in Bacteria, and is subdivided into box C/D and box H/ACA snoRNAs. In Eukarya, the majority of snoRNAs is located within introns of protein-coding genes and is processed by splicing followed by endo- and exonucleolytic cleavage [19],[23],[24].

Each member of the box C/D snoRNA family possesses characteristic sequence elements called box C (PuUGAUGA) and box D (CUGA), optional degenerate C′/D′ boxes and a short 5′-3′ terminal stem structure [24],[25]. 10–21 nt long sequence-specific antisense elements upstream of the boxes D/D′ guide the box C/D snoRNA core proteins fibrillarin, a RNA methyltransferase, Nop56, Nop58 and the 15.5 kD protein to the target RNA. 2′-O-methylation of the ribose at the fifth nucleotide upstream of the D/D′ box on the target RNA is carried out by the fibrillarin core protein [24]. Box H/ACA snoRNAs possess a distinctive common ACA sequence motif at their 3′-terminus and one to two stem-loop structures linked by a hinge (the so-called H-box motif: ANANNA, with N being any nucleotide), and guide the conversion of uridine to pseudouridine within the RNA target [26],[27]. The large number of conserved modifications in functionally conserved regions of rRNAs, such as the peptidyl-transferase centre, has suggested an important role for rRNA modifications in fine-tuning the structure and/or function of rRNAs [28]. It is important to note that a significant number of so-called “orphan” snoRNAs, lacking rRNA or snRNA targets, have been identified in Eukarya [29],[30]. However, the biological functions of orphan snoRNAs are still elusive.

In this study, we report, for the first time, the identification of a functional C/D box snoRNA within the EBV genome. We demonstrate that this viral snoRNA exhibits all bona fide box C/D snoRNA features with respect to its processing and expression, nucleolar localization as well as to canonical core protein binding partners. We also provide evidence that v-snoRNA1 is processed into a 24 nt long miRNA-like species which might target the 3′-UTR of the viral DNA polymerase mRNA.

## Results

### Identification of v-snoRNA1 by cDNA cloning and expression analysis

We have established an experimental strategy, designated as SHORT, to identify viral-induced ncRNAs in cord blood lymphocytes (CBL) infected with the EBV strain B95.8 [31]. The SHORT method is based on subtractive hybridisation of ncRNA populations of virus-infected cells from non-infected cells. NcRNAs, selectively expressed in the infected cell population, were subsequently converted into cDNAs. Sequencing of a small number, i.e. about 500 cDNA clones, allowed identification of several ncRNAs from the human as well as from the EBV genome whose expression was up-regulated upon viral infection [32].

**Figure 1:**
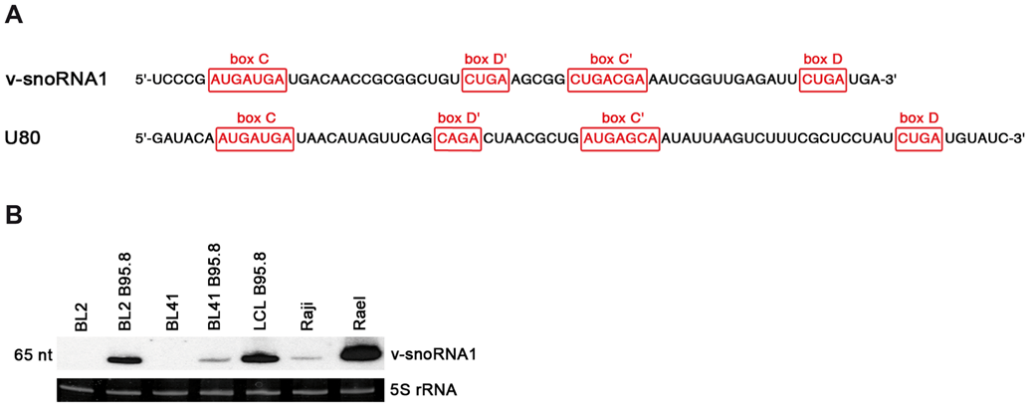
Sequence and expression profile of v-snoRNA1. (A) Sequences of the newly identified 65 nt long v-snoRNA1 and the canonical box C/D from snoRNA U80 used as a control are shown here. The position of C/D boxes and C′/D′ boxes are indicated in red. (B) Northern blot analysis showing expression of v-snoRNA1 in a panel of EBV-positive (BL2-B95.8, BL41-B95.8, LCL-B95.8, Raji, Rael) and EBV-negative (BL2, BL41) cell lines. 5S rRNA served as internal loading control.

Deep-sequencing analysis of 40.000 cDNA clones from this subtracted cDNA library further extended the list of differentially expressed ncRNAs (Hutzinger et al., manuscript in preparation). Interestingly, one of these sequences was represented by 95 cDNAs and exhibited all defining features of canonical C/D box snoRNA sequence motifs, i.e. C, C′, D′ and D boxes [24],[25]. Crucially, this potentially novel snoRNA species mapped to the EBV genome and was therefore designated as v-snoRNA1 (Figure 1A, and see above; Accession number FN376861). It is noteworthy that the canonical terminal stem-structure, formed by the 5′ and 3′ ends of eukaryal snoRNAs, was absent in the viral snoRNA, a feature shared with snoRNAs identified from archaeal or fungal species [33],[34].

To assess expression of v-snoRNA1, northern blot analysis was performed employing RNA from EBV-positive cell lines (Rael, Raji, BL2-B95.8, BL41-B95.8 and a LCL generated *in vitro* with the B95.8 virus) or EBV-negative cell lines (BL2 and BL41; Figure 1B). As expected, v-snoRNA1 could only be detected in infected cells but not in the EBV-negative control cells. Comparison with an internal RNA marker showed that the hybridized RNA species was 65 nt in size, which fully matched the size suggested by the original sequence obtained by cDNA cloning (see above and Figure 1B). Repeated attempts to identify v-snoRNA1-precursor transcripts by northern blot analysis were unsuccessful (unpublished data), suggesting that they are subjected to rapid processing.

**Figure 2:**
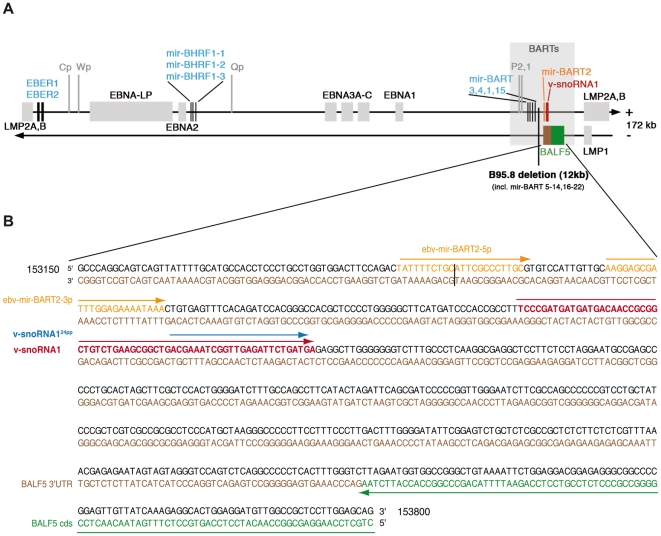
Schematic representation of the Epstein-Barr-virus genome. The location of ncRNA genes, latent genes and the precise location of v-snoRNA1 is indicated. (A) Location and transcription of EBV ncRNA genes (black lines with blue lettering) and EBV latent genes (grey bars with black lettering). The v-snoRNA1 is indicated in red, the neighboring miRNA BART2 in orange and the viral DNA polymerase BALF5 is depicted in green (for coding region) and brown (for 3′-UTR). The promoters are shown in grey lines and lettering, the BARTs region as a grey bar and the B95.8 deletion are also indicated. (B) Close-up of v-snoRNA1 location within the 3′-UTR of the viral DNA polymerase gene. The v-snoRNA1 is located on the sense strand about 60 nt downstream of the mir-BART2 precursor transcript and complementary to the BALF5 3′UTR that is situated on the antisense strand. V-snoRNA1^24pp^ is indicated in blue, other transcripts are indicated in the same colors as described above. The black line illustrates the cleavage site of mir-BART2. Corresponding EBV coordinates refer to the EBV B95.8 deletion strain (Accession number V01555.2).

The *v-snoRNA1* gene is located within the BamHI A rightward transcripts, known as BARTs, on the sense strand of the viral genome and maps about 100 nt downstream of the EBV mir-BART2 (Figures 2A and 2B). The BARTs represent abundant RNA species in EBV that are expressed in all latently infected EBV-B cell lines, in peripheral blood B cells of EBV-positive individuals and, at higher levels, in nasopharyngeal carcinoma [35],[36]. They do not encode for proteins but are processed into 22 different BART miRNAs (Figure 2A) [14]. Thereby, v-snoRNA1 as well as mir-BART2 arise from the same intron, which was found to be 4.9 kb in size in the AG876 strain (Accession number AJ507799) [35].

BART transcripts were previously shown to be predominantly transcribed from the P1 promoter [36]. However, P2 promoter-initiated BARTs were also detected in different B-cell lines with the exception of the EBV-positive BL cell line Raji. As shown in Figure 1B, v-snoRNA1 expression was verified in all tested EBV cell lines, including Raji cells, although expression levels varied considerably. In particular, v-snoRNA1 was expressed in Raji cells at barely detectable levels. Therefore, we infer that v-snoRNA1 transcription can be initiated at the P1 promoter but that the P2 promoter might be required to obtain full expression.

### Co-Immunoprecipitation and FISH analysis of v-snoRNA1

**Figure 3:**
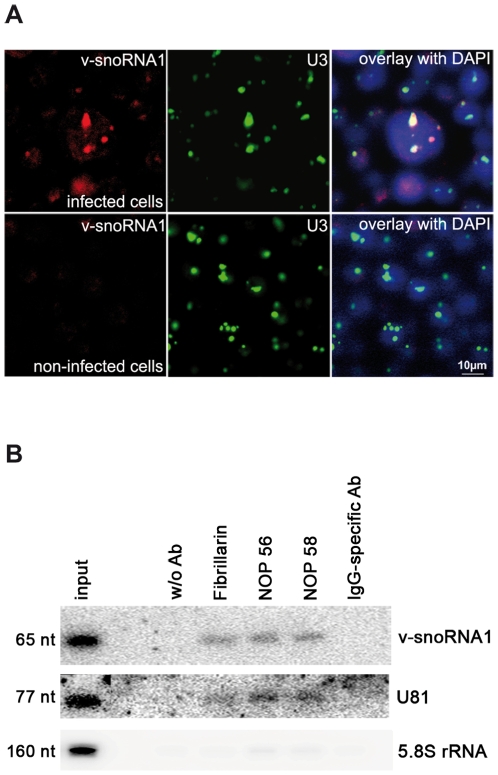
Fluorescent in situ hybridization and Co-immunoprecipitation of v-snoRNA1. (A) The box C/D v-snoRNA1 (red) localizes in the nucleolus of EBV-positive BL2-B95.8 cells. Box C/D snoRNA U3 (green) was used as a nucleolar marker. In EBV-infected cells both v-snoRNA1 and U3 co-localize in the nucleoli. In EBV-negative cells only U3 is expressed. The nucleus was stained with DAPI for visualization of nuclei and the scale bar is 10 µm. (B) Co-immunoprecipitation of v-snoRNA1 with fibrillarin, NOP56 and NOP58 snoRNP proteins. Following immunoprecipitation employing antibodies specific to fibrillarin, NOP56 and NOP58, the v-snoRNA1 was co-precipitated and detected via northern blot analysis. Box C/D snoRNA U81 and 5.8 rRNA were used as positive and negative controls, respectively.

To determine the sub-cellular location of v-snoRNA1 within EBV-infected cells, we employed fluorescent *in situ* hybridization (FISH) with dye-labeled antisense oligonucleotides complementary to v-snoRNA1. As a control, we also investigated the localization of U3 snoRNA, which is known to be localized in the nucleolus [37],[38]. Examination of EBV-infected BL2 cells by confocal microscopy revealed that both v-snoRNA1 and U3 snoRNA in fact co-localized to the nucleolus (Figure 3A). In contrast, a v-snoRNA1 hybridization signal was absent in non-infected B cells.

Canonical C/D box snoRNAs have previously been shown to bind to four snoRNA core proteins: fibrillarin, Nop56, Nop58, and the 15.5 K protein, respectively. These proteins have previously been shown to be strictly required for RNA maturation, stabilization and function [22],[39]. The C/D box proteins assemble with snoRNAs thus forming ribonucleo-protein complexes (snoRNPs) that localize to the nucleolus. In order to assess whether v-snoRNA1 assembles into a canonical C/D box snoRNP, binding of v-snoRNA1 to three of these canonical snoRNA-binding proteins (fibrillarin, Nop56 and Nop58) was assessed by co-immunoprecipitation using specific antibodies. Immuno-precipitated samples were subsequently analyzed for the presence of v-snoRNA1 by northern blot analysis. These assays demonstrated that v-snoRNA1 and the canonical U81 snoRNA, used as a positive control, were both co-immunoprecipitated with similar efficiencies with antibodies against all three snoRNA-binding proteins (Figure 3B). In contrast, none of the snoRNAs was precipitated in controls without antibodies or employing an IgG-specific antibody. Hybridization with an oligonucleotide specific for 5.8S rRNA was used to test for the specificity of the employed antibodies. Thereby, a faint, unspecific signal was detected in all samples after antibody addition, except the control without an antibody. This is likely caused by the high expression levels of 5.8S rRNA in our samples. From these results we conclude that the newly identified 65 nt long viral RNA transcript displays all hallmark features of a genuine box C/D snoRNA.

### v-snoRNA1 expression is strongly stimulated in the lytic cycle

**Figure 4:**
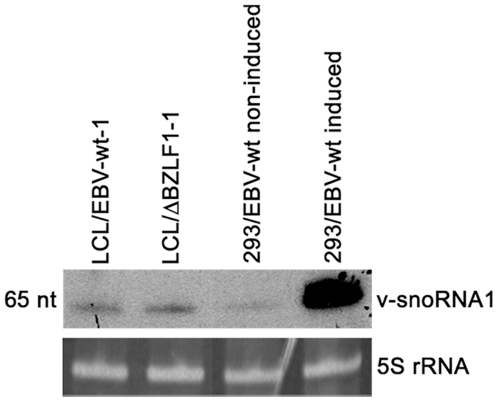
Expression of v-snoRNA1 during latency and lytic replication. Expression of v-snoRNA1 was investigated in LCLs infected with either the wild type or the replication-defective ΔBZLF1 EBV strain. The expression of v-snoRNA1 in 293 cells that stably carry the EBV-wt genome was monitored before and after induction with a BZLF1 expression plasmid. 5S rRNA was used as an internal loading control.

A common trait shared by all herpesviruses is their ability to infect their target cells under several modes; cells can support lytic replication during which new virus progeny is replicated or instead induce virus latency. Viral proteins used in both modes are usually, but not always, distinct. We therefore assayed v-snoRNA1 expression in latently infected cells or in cells undergoing lytic replication. We took advantage of LCLs established with viruses that are devoid of the lytic immediate early gene *BZLF1* (ΔBZLF1) and therefore cannot initiate lytic replication [40] and examined v-snoRNA1 expression in these cells by northern blot analysis (Figure 4). Northern blot signals were clearly visible in these cells thereby demonstrating that v-snoRNA1 is a latent transcript. We then performed the same experiment with replication-competent 293/EBV-wt cells lytically induced by transfection of the *BZLF1* gene (Figure 4). Comparison with non-induced cells showed that the v-snoRNA1 expression levels were up-regulated up to 30-fold following induction (Figure 4). V-snoRNA1 is therefore especially part of the EBV lytic expression programme.

### Phenotypic traits of a recombinant virus lacking v-snoRNA1

**Figure 5:**
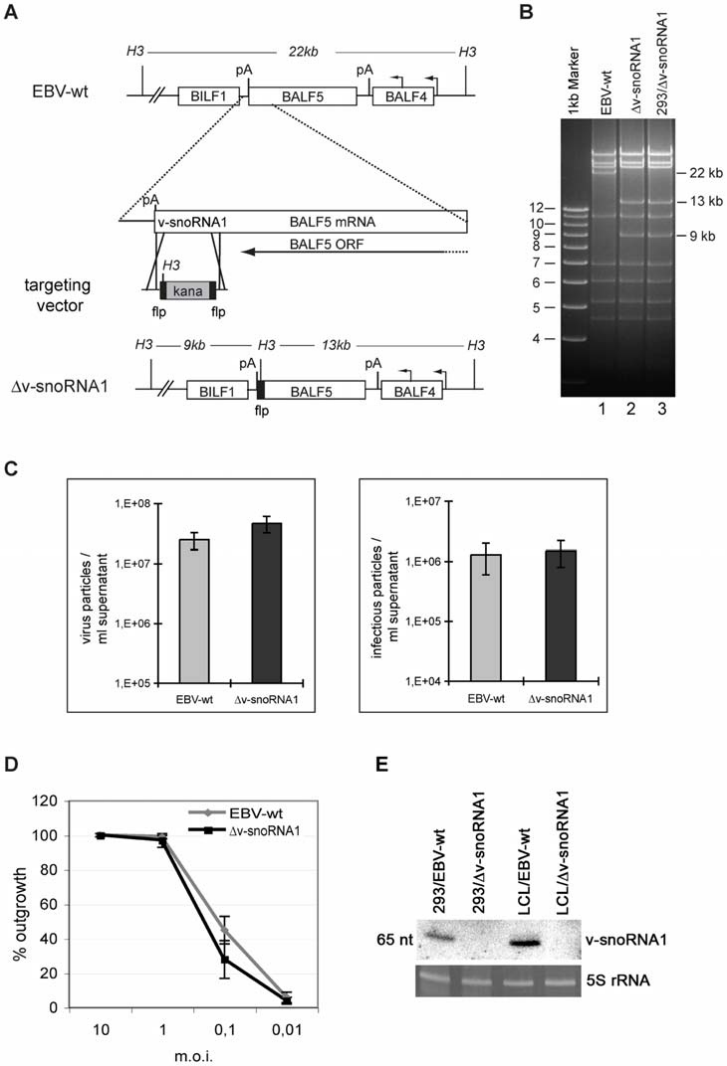
Construction of a v-snoRNA1 null recombinant virus. (A) Schematic map of the EBV genome segment that encompasses the v-snoRNA1 in EBV-wt before and after homologous recombination with the targeting vector carrying the kanamycin resistance gene flanked by flp recombinase recognition sites. The kanamycin cassette was excised in a second step. The restriction sites for HindIII (H3) and the expected fragment sizes after cleavage of EBV-wt and Δv-snoRNA1 genomes with this enzyme are given. pA: polyadenylation site, kana: kanamycin. (B) HindIII restriction fragment analysis of EBV-wt (lane 1) and Δv-snoRNA1 mutant genomes directly after construction in *E.coli* (lane 2) or after rescue from stably transfected 293 cells (293/Δv-snoRNA1) (lane 3). The result is fully consistent with the predicted restriction pattern (see A). (C) v-snoRNA1 is not required for virus production. Titres in supernatants from cells induced to produce viruses were determined either by measuring the concentration of viral genomes or by infecting the Raji B cell line in a limiting dilution assay. The concentration of viral genome equivalents and infectious particles is given for wild type and Δv-snoRNA1 viruses. Shown are mean values from three independent experiments. (D) Δv-snoRNA1 viruses show intact transforming properties. Primary B cells from three different normal donors were exposed to wild type and Δv-snoRNA1 viruses at various multiplicities of infection in a limiting dilution assay in cluster plates. The percentage of wells showing cell outgrowth is indicated. The presented results represent the average values from three experiments with the corresponding standard deviations. (E) v-snoRNA1 is expressed in cell lines infected with wild type EBV but not in cell lines infected with the Δv-snoRNA1 null-mutant. A northern blot analysis using a v-snoRNA1-specific probe was performed on 293 and B cells infected with either wild type EBV or with the Δv-snoRNA1-null mutant. 5S rRNA served as a loading control.

In an attempt to discover the function of v-snoRNA1 during the EBV life cycle, we constructed a recombinant virus that lacks a functional v-snoRNA1. To this aim, the C-box motif of v-snoRNA1 from the B95.8 strain was exchanged against the sequence of the kanamycin resistance gene flanked by two FLP recombinase recognition sites (Figure 5A). Excision of this cassette left an unrelated bacterial sequence containing a HindIII restriction site in place of the box C of v-snoRNA1 (Figure 5A and 5B, lane 2). DNA from the recombinant virus was stably transfected into 293 cells to generate a virus producer cell line, here referred to as 293/Δv-snoRNA1. Multiple clones were screened for their ability to support virus replication. One of the replication-competent clones was chosen at random for further experiments. Recombinant episomes purified from this producer cell line and transformed into *E. coli* cells were found to be intact as assessed by restriction analysis (Figure 5B, lane 3). Sequencing of the recombination site on these rescued episomes confirmed exchange of the Box C against unrelated DNA **TTTCCCG**CGCCAAGCTTCAAAAGCGCTCTGAAGTTCCTATACTTTCTAGAGAATAGGAACTTCGGAATAGGAACTTC**CAACC** (EBV DNA around the insertion is indicated in bold). A northern blot, performed on 293/Δv-snoRNA1 cells using a v-snoRNA1-specific probe, yielded negative results while signals could be clearly identified in the 293/EBV-wt positive control (Figure 5E, left panel). We therefore conclude that the Δv-snoRNA1 virus is devoid of the viral snoRNA and that destruction of the putative C box of v-snoRNA1 is sufficient to exert this effect.

We then conducted a series of experiments aiming at defining phenotypic traits of the mutant strain. We first assessed the ability of the 293/Δv-snoRNA1 to support viral replication. Viral titres were quantified either as packaged viral genome-equivalents (physical titres) or as green Raji units, i.e. as the concentration of viruses able to infect the Raji cell line determined by exposure to a limiting dilution of the viral supernatants (functional titres). Both assays revealed nearly identical titres for both the mutant and the wild type control (Figure 5D). The Δv-snoRNA1 viruses and producer cell line were then examined in electron microscopy; both displayed normal morphological features: encapsidation, primary and secondary egress were unchanged in the absence of the viral snoRNA (unpublished data). We further evaluated viral gene expression by western blot or immunostains (BZLF1, EA/D-BMRF1, gp350). Again, we could not discern any differences between the mutant and its wild type counterpart (unpublished data).

We then exposed various established cell lines or primary cells to the Δv-snoRNA1 mutant and monitored the efficiency of infection by counting the percentage of GFP-positive (293 cell line, primary epithelial cells) or EBNA2-positive (primary B cells) lymphocytes three days post-infection. The rate of infection was nearly identical in both wild type and mutant viruses (unpublished data). We finally investigated the transforming capacity of the mutant by performing infections of normal resting B cells from three different normal individuals at decreasing multiplicity of infections (Figure 5D). Wild type and mutant viruses both exhibited a transforming potential that resulted in a very similar number of outgrowing cell clones. We confirmed the identity of the viruses present in the growing LCLs by northern blot analysis; only LCLs generated by infection with wild type B95.8 virus expressed the snoRNA while those infected with Δv-snoRNA1 remained negative (Figure 5E, right panel).

### Computational and functional analysis of v-snoRNA1

**Figure 6:**
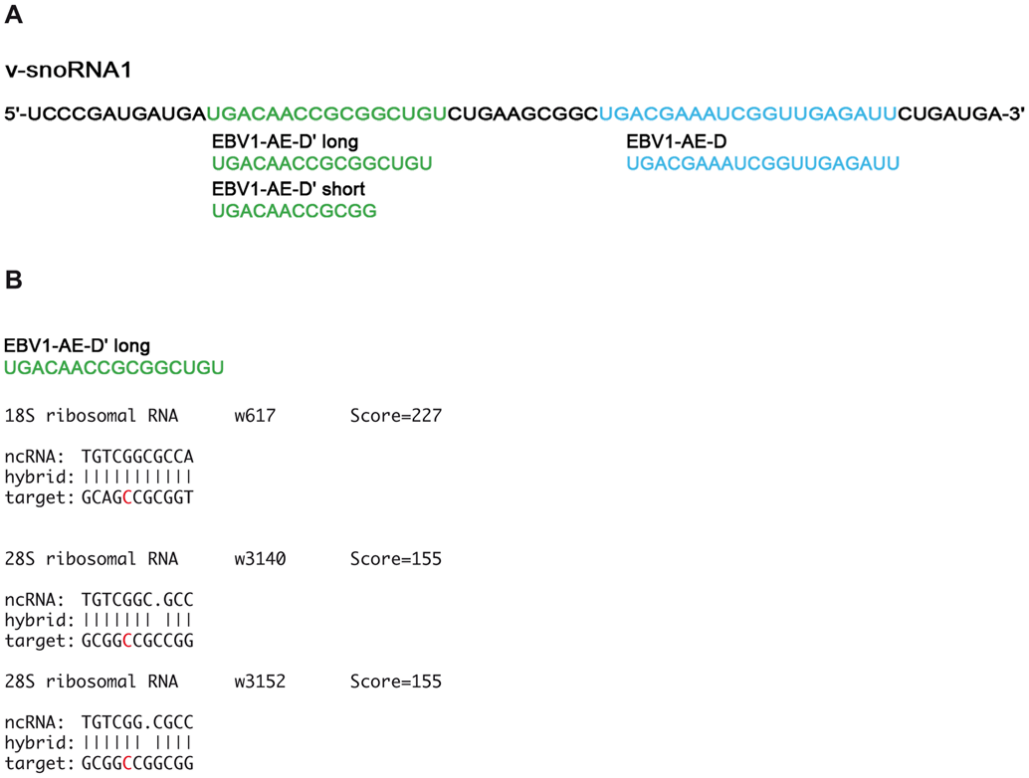
Sequence motifs of v-snoRNA1 antisense elements (AE) for computational target predictions. (A) AE box D is indicated in blue and the two potential alternative AE for box D′ in green. (B) A list of the most likely potential rRNA targets of AE box D′ long on the human genome is given (for the full list see Table S1). It includes the predicted ribose methylated positions (red), alignment and score.

The majority of snoRNAs have been found to target rRNAs or snRNAs by guiding ribose methylation or pseudouridinylation, respectively. In contrast, a number of snoRNAs lack telltale complementarities to canonical targets and hence are designated as “orphan” snoRNAs [19],[24],[30]. We therefore examined 18S and 28S rRNAs for putative v-snoRNA1 target sites using criteria established by Cavaille and Bachellerie [25]: the putative target sites were required to display at least a 7 nucleotides-long perfect complementarity with a region that ended within 3 nucleotides of the end of the snoRNA antisense boxes, and at most one nucleotide should be involved in a bulge or loop [25]. In particular we searched for putative target sites of the v-snoRNA1 box D antisense elements and for two potential alternative box D′ antisense elements (see Figure 6A). Using a program that was successfully used to predict targets of bacterial ncRNAs [41] we identified two putative ribose methylation site within the 18S rRNA and 23 sites within the 28S rRNA for box D′ (Table S1). However, none of the predicted target sites coincided with known methylated nucleotides within 18S and 28S rRNA. The same strategy applied to box D failed to reveal any putative ribose methylation sites within rRNAs. Nevertheless, we experimentally tested the ribose methylation status of the highest-scoring predictions for rRNA targets (Figure 6B) by primer extension analysis [42],[43]. However, no methylation at the predicted nucleotide positions C617 of human 18S rRNA and C3140 and C3152 of human 28S rRNA was observed in EBV-infected LCL B95.8 cells (data not shown), suggesting that v-snoRNA1 is a member of the still growing class of orphan snoRNAs.

### Processing of v-snoRNA1 into v-snoRNA1^24pp^: potential v-snoRNA1^24pp^ targets

**Figure 7:**
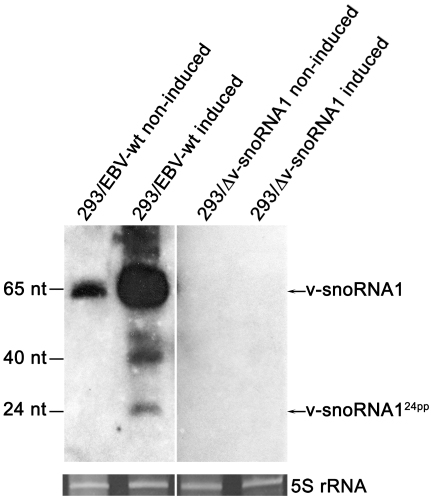
Expression analysis of v-snoRNA1^24pp^. Northern blot analysis demonstrating expression of the 24 nt long processing product v-snoRNA1^24pp^, derived from v-snoRNA1, by employing a specific LNA oligonucleotide probe in 293/EBV-wt or in 293/Δv-snoRNA1 knock-out strain cells without or upon *BZLF1*-induction. Expression of full length v-snoRNA1 (65 nt) and a potential cleavage intermediate (40 nt) are also shown. 5S rRNA serves as an internal loading control.

In addition to full-length cDNA clones encoding v-snoRNA1, we also identified nine identical partial cDNA clones of 24 nt in size in our cDNA library derived from the very 3′-end of v-snoRNA1 (Figure 2B). Previously, two studies were able to demonstrate processing of specific snoRNA species into functional miRNAs [44],[45]. Attempts to verify expression of the 24 nt long v-snoRNA1-derived processing product, designated as v-snoRNA1^24pp^, by northern blot analysis with conventional DNA oligonucleotide probes or by splinter ligation [44],[46] were unsuccessful (data not shown). In contrast, by applying a locked nucleic acid (LNA) probe, complementary to v-snoRNA1^24pp^, we were able to verify its expression (Figure 7). An additional hybridization signal at 40 nt was also observed that might represent a processing intermediate. All hybridization signals, except for full length v-snoRNA1, were only detected in the 293/EBV-wt cells induced with *BZLF1*, likely due to the high expression level of v-snoRNA1 within this strain. Notably, v-snoRNA1^24pp^ was not detected in the snoRNA knock-out strain (Figure 7).

**Figure 8:**
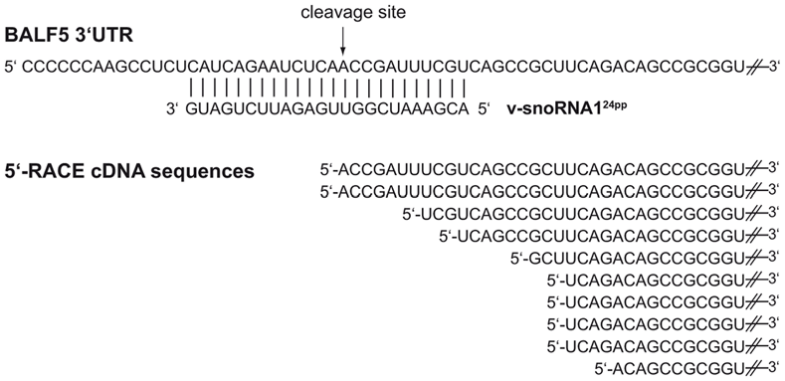
v-snoRNA1^24pp^-directed cleavage of BALF5 mRNA. The sequence of the 3′-UTR target site within the BALF5 mRNA and the complementary v-snoRNA1^24pp^ derived from v-snoRNA1 are shown. The predicted v-snoRNA1^24pp^-directed cleavage site according to cDNA sequences obtained from 5′-RACE is indicated by an arrow. cDNA sequences from 5′-RACE are shown at the bottom.

Since the 3′-UTR of the BALF5 mRNA exhibits full complementarity to v-snoRNA1^24pp^ (Figure 8) we investigated whether it might serve as a potential target site for cleavage by applying a 5′-RACE approach, as previously described [47],[48]. 5′-RACE products from the predicted 3′-UTR cleavage site were amplified by specific primers and sequenced (Figure 8). Indeed, we detected two clones corresponding exactly to a predicted cleavage site by v-snoRNA1^24pp^ 11 nt from its 5′-end in 293/EBV-wt cells induced with *BZLF1* which exhibits highest expression levels of v-snoRNA1 (Figures 4 and 7). Remaining clones from this region exhibited shorter sequences likely due to exonucleolytic degradation of the BALF5 mRNA following initial cleavage by v-snoRNA1^24pp^ as described previously for plant miRNAs [47]. Notably, not a single sequence was observed that was longer than the expected size, which is indicative of a specific cleavage event triggered by v-snoRNA1^24pp^ and followed by exonucleolytic degradation. In contrast, no fragments cleaved within the 3′-UTR of BALF5 mRNA were observed in the snoRNA knock-out strain.

### Conservation of v-snoRNA1 in other viral genomes

**Figure 9:**
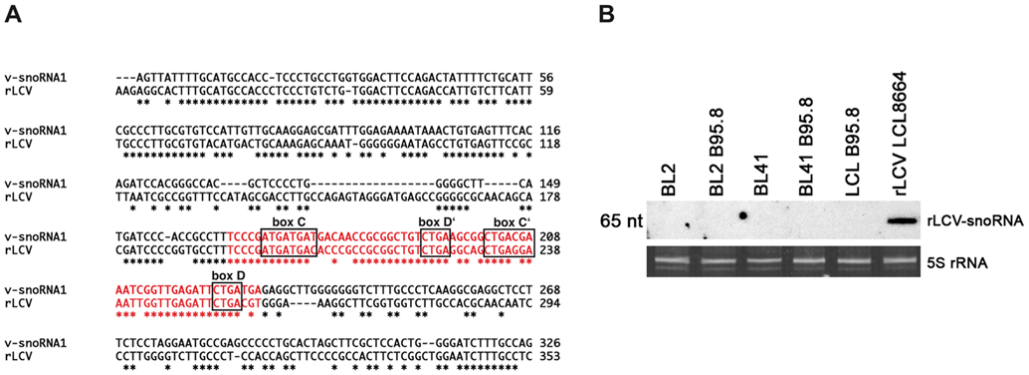
Expression of v-snoRNA1 in rLCV. (A) Alignment of v-snoRNA1 and rLCV including their flanking regions. V-snoRNA1 and rLCV snoRNA sequences are marked in red, flanking nucleotides in black. Stars in red and black indicate conservation of the nucleotides between EBV and rLCV sequences. The boxes C/D and C′/D′ are encircled by black rectangles. (B) The putative rLCV snoRNA is expressed in simian LCLs. A northern blot analysis using a labeled rLCV snoRNA oligonucleotide was performed on the LCL8664 cell line that was generated by infection with rLCV. A panel of EBV-negative and EBV-positive human LCLs were used as controls. 5S rRNA was used as loading control.

The identification of a snoRNA species in a viral genome raised two obvious questions: is v-snoRNA1 conserved among the different herpesvirus subfamilies or even among several EBV strains and do v-snoRNA1 homologs exist in other virus families? This prompted us to perform a BLAST alignment search using all available databases. This search showed that the v-snoRNA1 sequence is 100% conserved among the tested EBV strains (B95.8, AG876, M81, GD1, Raji). It further revealed that the distantly related rhesus lymphocryptovirus (rLCV) genome (exhibiting an overall sequence identity of 65% with the EBV genome; Accession number NC_006146) contains a 65 base pair sequence that shows 86% identity with v-snoRNA1 (Accession number FN376863). In particular, the canonical D, D′ and C, C′ boxes were universally conserved as well as antisense elements, preceding D or D′ boxes. This high degree of sequence identity did not extend to the v-snoRNA1 flanking regions; these showed only 69% sequence identity and were therefore clearly less conserved (Figure 9A). Northern blot analysis, employing an rLCV-specific antisense oligonucleotide, confirmed that the rLCV sequence homolog of v-snoRNA1 is actively transcribed and processed into an RNA species of 65 nt in simian B cells (Figure 9B). Despite the high degree of sequence identity between human and rLCV v-snoRNA1s, hybridization with the rLCV-specific probe did not detect its EBV counterpart. Altogether, these findings strongly indicate that rLCV also encodes a box C/D snoRNA homolog to v-snoRNA1.

## Discussion

Herpes virus genomes carry numerous cellular gene homologs [49]. Many of these genes encode house keeping proteins but others serve more specialized functions e.g. within the host immune system. This is particularly true of γ-herpesviruses whose genomes encode homologs of cytokines (e.g. CSF-1 and IL10 for EBV, IL6 for Kaposi's sarcoma-associated herpesvirus (KSHV) or of anti-apoptotic mediators (e.g. BCL2 in EBV and KHSV). These striking homologies between a virus and a cellular genome were reinforced by the discovery that herpesviruses encode multiple miRNA clusters. Here we report that herpesviruses and their host share yet another fundamental ncRNA species.

Deep-sequencing analysis of a subtracted cDNA library that was constructed to specifically identify transcripts expressed in EBV-infected B cells allowed discovery of a viral transcript that exhibited all defining features of a C/D box snoRNA. Indeed, v-snoRNA1 comprises canonical C/C′ as well as D/D′ boxes. It is of note that v-snoRNA1 is lacking the canonical terminal stem-structure usually encountered in eukaryal snoRNAs. In this respect, v-snoRNA1 appears to be closer to snoRNA species previously identified in fungi or in the domain of Archaea [33],[50]. In addition to the EBV-encoded v-snoRNA1, the genome of the Herpesvirus saimiri (HVS), a member of the γ-herpesvirus family, was recently reported to encode seven small nuclear RNAs [51],[52]. Thereby, in latently infected HVS-transformed T cells, the Herpesvirus saimiri U RNAs (HSURs) represent the most abundant viral transcripts. Similar to EBERs, HSURs are not essential for viral replication or transformation, but are involved in the activation of specific genes in virus-transformed T cells during latency [51].

V-snoRNA1 was found to be expressed in all samples of a panel of EBV-positive cell lines that included several BLs and in particular the latency I Rael cell line, LCLs and the 293/EBV-wt producer cell line (Figure 1). Detection of reduced levels of v-snoRNA1 in LCLs, generated with the BZLF1-null virus that therefore cannot undergo lytic replication, demonstrated that v-snoRNA1 is an integral part of the EBV latent transcription program (Figure 4). However, expression levels of v-snoRNA1 increased significantly up to 30-fold upon induction of the lytic replication cycle. This is consistent with a model that v-snoRNA1 serves, presumably different, functions in both the latent and the lytic mode of infection.

Three findings demonstrated that v-snoRNA1 is likely to represent a fully functional ncRNA species. V-snoRNA1 was found to co-localize with canonical snoRNA to the nucleolus (Figure 3). Furthermore, we could show that v-snoRNA1 assembles into a canonical snoRNP that at least includes the fibrillarin, Nop56 and Nop58 proteins. Finally, selective destruction of the C box resulted in a complete down-regulation of steady state levels of v-snoRNA1 (Figure 5E). This is consistent with previous work that ascribed an essential role in the regulation of the stability of snoRNA to this sequence motif [21],[53],[54].

V-snoRNA1 could be localized to the BARTs region which follows a complex splicing pattern and also encodes a cluster of non-coding miRNA genes (Figure 2). V-snoRNA1 was located outside the putative BARTs open reading frame and is therefore, as previously observed for canonical eukaryal snoRNAs, likely processed from an intron. The BARTs transcripts can be initiated from two promoters P1 and P2 [36]. Analysis of v-snoRNA1 expression levels showed a large degree of variation within the tested cell lines, as was also observed for EBV's miRNAs [55]. In principle, this could be related to the highly variable virus copy numbers among different EBV-positive cell lines. Alternatively, it may be related to the propensity of some of these cell lines to undergo lytic replication. The low expression levels of v-snoRNA1 in Raji are probably due to an inactive BART P2 promoter; this suggests that the P2 promoter initiates most of the v-snoRNA1 transcripts.

The discovery of a snoRNA in a Herpesvirus genome prompted us to search for homologs in other viral or cellular genomes. This search revealed that the v-snoRNA1 is strictly conserved across five distinct EBV strains. It further led to the identification of a transcript within the rLCV genome that displays a high degree of homology to v-snoRNA1. This genetic element comprises perfectly conserved canonical C/D and C′/D′ boxes and was expressed in a simian LCL which suggests that rLCV also encodes a snoRNA. Discovery of a v-snoRNA1 homolog in rLCV is not entirely unexpected; rLCV is the closest EBV relative as both genomes exhibit 65% sequence identity and, therefore, display more than 80% sequence identity for protein-coding genes and ncRNA genes. Indeed, seven rLCV miRNA were found to be closely related to their EBV counterparts [11]. The relatively crude approach (BLAST) we initially took failed to reveal further v-snoRNA1 relatives; we nevertheless consider that this question is still open and hope that our work will stimulate research in this direction.

The strict conservation of v-snoRNA1 domains within various EBV strains and among evolution strongly suggests that this element serves an essential role in the natural history of EBV infection. We therefore initiated a series of experiments that aimed at defining potential functions of v-snoRNA1. We thereby combined a computational with an experimental approach to determine putative ribosomal or spliceosomal RNA targets for v-snoRNA1 using previously identified criteria (see results section). However, both attempts failed to identify any obvious rRNA candidates. Hence, v-snoRNA1 can be assigned in all probability to the class of so-called “orphan” snoRNAs that lack rRNA or snRNA targets (see below).

Another strategy to discover the function of v-snoRNA1 consisted in constructing a v-snoRNA1-null mutant and defining its phenotypic traits using well-characterized *in vitro* assays. As of now, the Δv-snoRNA1 mutant remained indistinguishable from its wild type counterparts in terms of lytic replication, infection and B cell transformation (Figure 5). However, this does not exclude that v-snoRNA1 serves an important function during the virus life cycle; unraveling miRNAs contributions to EBV infection has also proven a difficult enterprise. Aside from a few notable exceptions such as miR-BART5 and miR-BART2 that respectively target the cellular gene *PUMA* [56] and the viral gene *BALF5* [57] or the BART cluster 1 and BHRF1-2 that respectively modulate LMP1 expression and BHRF1 mRNA processing [58],[59], the essential functions served by these ncRNAs remain unclear. Indeed, the B95.8 strain that lacks a large number of miRNAs perfectly replicates and immortalizes primary B cells with high efficiency.

Recently, specific snoRNA species have been characterized as miRNA precursors, which are processed to mature miRNAs and assemble into a functional RNA induced silencing complex [60],[61]. Indeed, by deep-sequencing we identified nine identical cDNA clones of 24 nt in size, that mapped to the very 3′-end of v-snoRNA1. The expression of v-snoRNA1^24pp^ was verified by northern blot analysis employing a specific LNA oligonucleotide antisense probe (Figure 7). Thereby, the hybridization signal was especially apparent in 293/EBV cells induced by *BZLF1*, which results in a 30-fold up-regulation of v-snoRNA1 expression; the hybridization signal was absent, however, in non-induced wild type cells. This could be explained by lower v-snoRNA1 expression levels in non-induced 293/EBV cells, compared to *BZLF1*-induced cells (Figure 7), resulting in reduced processing of v-snoRNA1^24pp^ below the northern blot detection limit. Alternatively, this finding could result from preferential processing of v-snoRNA1 into v-snoRNA1^24pp^ during lytic replication.

Subsequently, by a 5′-RACE approach we also investigated a potential target for snoRNA1^24pp^. Since the RNA species maps in antisense orientation to the 3′-UTR of the BALF5 mRNA, which encodes the viral DNA polymerase, BALF5 mRNA might represent a likely target site. As has been shown previously, the 3′-UTR of the BALF5 mRNA encodes in antisense orientation, in addition to v-snoRNA1^24pp^, a bona fide EBV miRNA, designated as mir-BART2. Thereby, it has been reported that mir-BART2 down-regulates the mRNA levels by cleavage within the BALF5 3′-UTR [57]. According to the proposed model, mir-BART2 thereby inhibits the transition from latent to lytic viral replication. By 5′-RACE analysis, we provide evidence that v-snoRNA1^24pp^ might also target BALF5 mRNA for cleavage and subsequent degradation. In contrast to mir-BART2, however, expression of v-snoRNA1^24pp^ was only apparent upon induction of the viral lytic cycle by *BZLF1* (Figure 7). Future experiments will focus on the function of v-snoRNA1 and v-snoRNA1^24pp^ especially in respect to its function in the latent and lytic cycles of EBV infection.

## Materials and Methods

### Cell lines and EBV strains

The cell lines BL2, BL2 B95.8, BL41, BL41 B95.8, CBL B95.8, LCL B95.8, Raji, Rael, HEK293 and LCL8664 were cultured in RPMI 1640 supplemented with 10% FCS, L-glutamine (2 mM ml^−1^) and antibiotics (100 U penicillin ml^−1^ and 100 µg streptomycin ml^−1^). BL2 and BL41 are EBV-negative Burkitt's Lymphoma cell lines, BL2 B95.8 and BL41 B95.8 are cell lines infected with EBV strain B95.8, Raji and Rael are EBV-positive Burkitt's lymphoma cell lines [62],[63]. CBL B95.8 and LCL B95.8 cells were obtained after *in vitro* transformation of cord blood lymphocytes (CBL) or primary human blood lymphocytes (LCLs) with the B95.8 strain of EBV. The EBV deletion strain B95.8, used in this study, lacks a 12 kb large portion of the genome [64]. LCL8664 is a rhesus LCV (cercopithicine herpesvirus 15)-infected B-cell line derived from a retro-orbital B-cell lymphoma in a rhesus monkey [65]. 293/EBV-wt contains the wild type EBV B95.8 genome in a recombinant form. Two LCL/EBV-wt and LCL/ΔBZLF1 pairs were established by immortalization of B cells from two different donors with either wild type or BZLF1-negative recombinant viruses [40].

### RNA preparation and northern blot analysis

Total RNA from EBV-infected and non-infected cells was isolated by using the Tri Reagent method according to the manufacturers protocol. Northern blot analysis was performed as described in Mrazek et. al [32] applying a mix of five oligonucleotides (F1: CCTCTCATCAGAATCTCAACC, F2: TCTCAACCGATTTCGTCAGC, F3: CGTCAGCCGCTTCAGACAG, F4: GACAGCCGCGGTTGTCATC, F5: GGTTGTCATCATCATCGGGAA) covering the whole v-snoRNA1 sequence. For the detection of the homologous rhesus lymphocryptovirus a rLCV-specific v-snoRNA1 oligonucleotide (5′-AATCTCAACCAATTTCCTCAGC-3′) was used. Detection of v-snoRNA1^24pp^ by an LNA oligonucleotide (5′-CATCAGAATCTCAACCGATTTCGT-3′, Exiqon) was performed according to the standard protocol, except 60 µg RNA was loaded and membrane was washed under stringent conditions. 5.8S rRNA antisense oligonucleotide 5′-TCCTGCAATTCACATTAATTCTCGCAGCTAGC-3′ was used as negative control in immunoprecipitations. Ethidium bromide-stained 5S rRNA were used as loading control for normalization after polyacrylamid gel electrophoresis. Northern blot signals were either put onto Kodak MS-1 film, using an intensifier screen or analyzed with a Molecular Dynamics Storm PhosphorImager (Image quant software version 5.0).

### Fluorescent in situ hybridization

For the detection of the viral and U3 snoRNA the following amino-modified DNA oligonucleotides were used:AT*CTCAACCGATT*TCGTCAGCCGCT*TCAGACAGCCGCGGT*TGTCATCAT*CAT for v-snoRNA1 and GT*TCTCTCCCTCT*CACTCCCCAAT*ACGGAGAGAAGAACGAT*CATCAATGGCT*G for U3 (the amino-modified T nucleotides are marked with asterisks). The probes were labeled with CY3 (v-snoRNA1; Amersham Biosciences) or Oregon Green 488 (U3; Molecular Probes) according to the manufacturers protocol.

BL2 and BL2-B95.8 cells were washed in 1× PBS (PBS: 100 mM Na_2_HPO_4_, 20 mM KH_2_PO_4_, 137 mM NaCl, 27 mM KCl, pH 7.4) and diluted in 1× PBS to an appropriate concentration. The cell suspension was dropped onto glass slides and hybridized according to [21],. The slides were washed 3 times for 20 min after hybridization and mounted with 15 µl Mowiol containing 0.1 µg/ml DAPI. Slides were analyzed by confocal fluorescence microscopy (LSM 510 META, Carl Zeiss GmbH) using Zeiss LSM Software, version 3.2.

### Co-Immunoprecipitation

For the preparation of snoRNP extract, BL2-B95.8 cells were washed in 1× PBS, lysed in 5-fold amount of 1× RNP lysis buffer (25 mM Tris-HCl pH 7.5, 150 mM NaCl, 0.2 mM EDTA pH 8.0, 0.2% TritonX-100), sonicated and incubated for 1 h on ice. After centrifugation steps at 18000×g, 4°C, 10 min and 30000×g, 4°C, 30 min the snoRNP extract was used for co-immunoprecipitation.

250 µl Protein A/G PLUS-Agarose (Santa Cruz Biotechnology Inc.) was washed three times in 1× PBS and resuspended with 1× RNP lysis buffer to receive a final volume of 125 µl. 50 µl of the suspension was added to the total cell lysate containing 500 µg protein extract for each approach and precleared for 1 h at 6°C during rotation. The precleared supernatant was equally distributed and incubated with specific antibodies for fibrillarin (ab5821; Abcam), NOP56, NOP58, and IgG (Santa Cruz Biotechnology Inc.) for 1 h at 6°C. After addition of 12 µl of washed beads to each approach and rotation for 4 h at 6°C, samples were centrifuged at 800×g at 4°C for 5 min. The supernatant was used as a control for unbound RNA and the remaining beads were washed four times with 1 ml 1× RNP lysis buffer and the co-immunoprecipitated RNA was eluted with 200 µl IP elution buffer (100 mM Tris-HCl pH 7.5, 150 mM NaCl, 12.5 mM EDTA pH 8.0, 20% SDS) after heating for 5 min at 95°C. The supernatant was used to perform phenol-chloroform-isoamylalcohol (Fluka) extraction, RNA was ethanol-precipitated over night and analyzed by northern blot analysis.

### Recombinant EBV genomes

The wild type EBV recombinant plasmid (p2089) is cloned onto the prokaryotic F factor origin of replication and carries the green fluorescent protein (gfp), the chloramphenicol (cam) resistance gene and the hygromycin (hyg) resistance gene [67]. The EBV snoRNA mutant was constructed by replacing the C box sequence motif (B95.8 coordinates 153331–153341) with the kanamycin (kan) resistance gene using homologous recombination [68]. Composite primers were used whose internal parts (underlined) are specific for the kan resistance gene, and whose external parts (40 bp) are specific for the snoRNA gene (5′-ACGCTCCCCTGGGGGCTTCATGATCCCACCGCCTTTCfCCGCGCCAAGCTTCAAAAGCGCTC-3′; 5′-CTCAACCGATTTCGTCAGCCGCTTCAGACAGCCGCGGTTGGAAGTTCCTATTCCGAAGTTCC-3′).

These primers allowed PCR-mediated amplification of the kan resistance gene through their internal sequences and then homologous recombination of the amplified PCR product with the EBV wild type genome via their external sequences. PCR amplification products were incubated with the restriction enzyme DpnI to remove traces of the parental plasmid and introduced by electroporation (1000 V, 25 µF, 100 Ω) into *E. coli* DH10B cells carrying the recombinant virus p2089 and the temperature sensitive pKD46 helper plasmid encoding the phage lambda red recombinase to foster homologous recombination. Cells were grown in LB with cam (15 µg/ml) at 37°C for an hour and then plated onto LB agar plates containing cam (15 µg/ml) and kan (10 µg/ml). Incubation at 42°C induced the loss of the helper plasmid. DNA of positive clones was purified and analyzed with HindIII restriction enzyme to confirm correct recombination. The kan resistance gene was excised using the Flp recombinase cloned onto the temperature-sensitive plasmid pCP20 [69] which also carries the amp resistance gene. The bacterial clones that resulted from selection on cam/amp plates were further grown on cam plates at 42°C to induce the loss of the pCP20 plasmid. Resistant clones were then submitted to restriction analysis to confirm the expected restriction pattern. Sequencing further confirmed successful recombination and the intactness of the flanking regions.

### Stable clone selection

HEK293 cells were transfected with the properly recombined mutant viral DNA (clone B 253) using Lipofectamine (Invitrogen) as described [70]. Selection of stable 293 cell clones carrying the EBV recombinant plasmid was performed by addition of hygromycin to the culture medium (100 µg/ml). Cell clones surviving selection were first assessed for GFP fluorescence and the positive clones were further expanded. Fifteen clones were assessed for their ability to support lytic replication by qPCR. Ten of those were found to produce virus at high levels, one of which was selected for further analysis. The cell clone used in this study is referred to as 293/Δv-snoRNA1. Viral episomes from this clone were transferred back in E.coli and submitted to restriction analysis and sequencing.

### Plasmid rescue in *E. coli*

Circular plasmid DNA from 293/ΔsnoRNA and was extracted using a denaturation-renaturation method as described previously [71]. *E.coli* strain DH10B was transformed with the viral recombinant DNA by electroporation as described before [68] and clones were selected on LB plates containing cam (15 µg/ml). Single bacterial colonies were expanded and DNA plasmid preparation submitted to digestion with restriction enzyme HindIII.

### Virus induction and infection of target cells

Producer cell clones 293/EBV-wt (carrying p2089) and 293/Δv-snoRNA1 were transfected with a BZLF1 (Accession number NC_007605.1) expression plasmid (0.5 µg/well) to induce lytic cycle [72] using lipid micelles (Metafectene, Biontex) according to manufactures instructions. Virus supernatants were harvested four days post transfection, filtered through a 0.8 µm filter and stored at −80°C. Viral titers were determined by infecting 10^4^ Raji cells with increasing dilutions of EBV-wt or Δv-snoRNA1 supernatants. Three days after infection, gfp-positive Raji cells were counted using a fluorescent microscope (Leica). For immortalization assays, primary B cells were mixed with infectious supernatants at various multiplicities of infections (MOI) and seeded into U-bottom 96-well plates coated with gamma-irradiated WI38 feeder cells [73] at a concentration of 10^2^ cells per well. Wells containing outgrowing LCL clones were counted.

### Quantitative real-time PCR

Detection of viral DNA and calculation of viral titers was carried out by quantitative real-time PCR (qPCR) using BALF5-specific primers and probe as described [74]. The DNA content was calculated using a serial dilution of Namalwa DNA, a human Burkitt's lymphoma cell line that contains two EBV genome copies per cell, as a standard curve.

### Computational prediction of target sites in rRNAs

We predicted putative rRNA target sites for the snoRNAs in this study as follows. We first downloaded from Genbank the sequences of the human 18S (Accession NR_003286) and 28S (Accession NR_003287) rRNAs. The sequences of the antisense D-box (TGACGAAATCGGTTGAGATT) and D′-box (TGACAACCGCGGCTGT) were used to search for subsequences with good complementarity to the rRNAs with the program described in Mandin P et al. [41]. As the study of Cavaille & Bachellerie [25] indicated that snoRNA-rRNA interactions involve regions of at least 7 nucleotides complementarity that are located at most 3 nucleotides from the end of the snoRNA antisense box, and that bulges and loops of more than 1 nucleotide are disfavored, we implemented these constraints in our programs. That is, we first used relatively large penalties for the introduction and extension of bulges and loops (a score penalty of 8), and we restricted the maximum size of loops and bulges to 1 nucleotide. The energy parameters of nucleotide-nucleotide interactions were kept with their default values coded in the program. We then extracted only hybrids that contained at least 7 nucleotide-nucleotide pairs, that ended within 3 nucleotides of the end of the antisense box, and that did not contain more than one bulge or loop.

### Primer extension assay

2-OH ribose methylation of rRNA was assayed as follows. Oligonucleotides (0.6 pM) were 5′-end-labeled with ^32^P-γ-ATP and heat-denaturated after addition of 3 µg of total RNA (LCL B95.8) for 2 min at 96°C. Primer annealing was performed in presence of 30 mM KCl and 25 mM Tris-HCl pH 8.4 for 30 min at 42°C. Reverse transcription was carried out for 45 min at 42°C in buffer containing 100 mM Tris/HCl pH 8.4, 10 mM MgCl_2_, 15 mM KCl, 10 mM DTT, 0.5/0.02/0.005 mM dNTPs and 0.4 U AMV reverse transcriptase. Additionally, a final concentration of 0.0625 mM dideoxynucleotides was added to the sequencing reactions. The reactions were stopped by addition of twice volume of 4 M NH_4_Ac and 20 mM EDTA, cDNA products were precipitated, resolved on a 10% denaturating polyacrylamide gel and visualized by autoradiography.

### 5′ RACE of the viral *BALF5*

Total RNA of 293/Δv-snoRNA1 and 293/EBV-wt induced by *BZLF1* and was adaptor-ligated and reverse transcribed using a gene-specific primer (5′- TTCGCCCTTGCGTGTCCATTGT-3′) according to the FirstChoice RLM-RACE Kit (Ambion). cDNA was PCR-amplified with the non-specific 5′ RACE outer primer and the same reverse primer and further amplified by nested PCR using the 5′ RACE inner primer and a second gene-specific reverse primer (5′- GCAAGGAGCGATTTGGAGAAAATAAAC-3′). PCR DNA was gel purified, cloned (pGEM-T Easy Vector System I, Promega) and subjected to Sanger sequencing employing the ABI Prism 3100 capillary sequencer (Perkin Elmer).

### Accession numbers

v-snoRNA1: FN376861; BZLF1: NC_007605.1; 18S rRNA: NR_003286; 28S rRNA: NR_003287; Epstein-Barr-Virus genome, strain AG876: AJ507799; Rhesus lymphocryptovirus genome: NC_006146

## Supporting Information

Table S1 Potential target sites of 18S and 28S rRNA complementary to v-snoRNA1 AE D′.
(0.05 MB PDF)
