# Supplementary material for: Expression and Processing of a Small Nucleolar RNA from the Epstein-Barr Virus Genome
Source: PLoS Pathog. 2009 Aug 14;5(8):e1000547. doi: 10.1371/journal.ppat.1000547 (PMC2718842; doi:10.1371/journal.ppat.1000547)
Supplement: Table S1 — Potential target sites of 18S and 28S rRNA complementary to v-snoRNA1 AE D′. (0.05 MB PDF) [file ppat.1000547.s001.pdf]

Table S1. Potential target sites of 18S and 28S rRNA complementary to v-snoRNA1 AE D'.

```
>EBV1-box-D
TGACGAAATCGGTTGAGATT

>EBV1-box-D' long
TGACAACCGCGGCTGT

>EBV1-box-D'short
TGACAACCGCGG


18S target predictions: box-D' long

#>EBV1-box-D'16

>Homo_sapiens_18S_ribosomal_RNA-w617    Score=227    ncRNA_start=6ncRNA_end=16
target_start=617    target_end=627
ncRNA:       TGTCGGCGCCA
hybrid:      |||||||||||
target:      GCAGCCGCGGT

>Homo_sapiens_18S_ribosomal_RNA-w873    Score=131    ncRNA_start=5ncRNA_end=14
target_start=873    target_end=883
ncRNA:       TC.GGCGCCAA
hybrid:      || ||||||||
target:      GGACCGCGGTT


28S target predictions: box-D' short

#>EBV1-box-D'short  12

>Homo_sapiens_28S_ribosomal_RNA-w2688   Score=130    ncRNA_start=5ncRNA_end=12
target_start=2688   target_end=2695
ncRNA:       GGCGCCAA
hybrid:      ||||||||
target:      TCGCGGTT


28S target predictions: box-D' long

#>EBV1-box-D'16
>Homo_sapiens_28S_ribosomal_RNA-w3536   Score=181    ncRNA_start=5ncRNA_end=14
target_start=3536   target_end=3545
ncRNA:       TCGGCGCCAA
hybrid:      ||||||||||
target:      GGCCGCGGTT

>Homo_sapiens_28S_ribosomal_RNA-w3498   Score=156    ncRNA_start=7ncRNA_end=14
target_start=3498   target_end=3505
ncRNA:       TCGGCGCC
hybrid:      ||||||||
target:      GGCCGCGG
```

```
>Homo_sapiens_28S_ribosomal_RNA-w3140   Score=155    ncRNA_start=7ncRNA_end=16
target_start=3140   target_end=3150
ncRNA:       TGTCGGC.GCC
hybrid:      ||||||| |||
target:      GCGGCCGCCGG

>Homo_sapiens_28S_ribosomal_RNA-w3152   Score=155    ncRNA_start=7ncRNA_end=16
target_start=3152   target_end=3162
ncRNA:       TGTCGG.CGCC
hybrid:      |||||| ||||
target:      GCGGCCGGCGG

>Homo_sapiens_28S_ribosomal_RNA-w2926   Score=149    ncRNA_start=7ncRNA_end=16
target_start=2926   target_end=2934
ncRNA:       TGTCGGCGCC
hybrid:      || |||||||
target:      GC.GCCGCGG

>Homo_sapiens_28S_ribosomal_RNA-w4704   Score=149    ncRNA_start=7ncRNA_end=16
      target_start=4704   target_end=4712
ncRNA:       TGTCGGCGCC
hybrid:      || |||||||
target:      GC.GCCGCGG

>Homo_sapiens_28S_ribosomal_RNA-w2685   Score=142    ncRNA_start=5ncRNA_end=16
target_start=2685   target_end=2695
ncRNA:       TGTCGGCGCCAA
hybrid:      || |||||||||
target:      GC.GTCGCGGTT

>Homo_sapiens_28S_ribosomal_RNA-w845    Score=138    ncRNA_start=6ncRNA_end=16
target_start=845    target_end=855
ncRNA:       TGTCGGCGCCA
hybrid:      ||||| |||||
target:      GCGGCGGCGGT

>Homo_sapiens_28S_ribosomal_RNA-w2275   Score=138    ncRNA_start=6ncRNA_end=16
target_start=2275   target_end=2285
ncRNA:       TGTCGGCGCCA
hybrid:      || ||||||||
target:      GCCGCTGCGGT

>Homo_sapiens_28S_ribosomal_RNA-w3273   Score=138    ncRNA_start=9ncRNA_end=16
target_start=3273   target_end=3280
ncRNA:       TGTCGGCG
hybrid:      ||||||||
target:      GCGGCCGC

>Homo_sapiens_28S_ribosomal_RNA-w3372   Score=138    ncRNA_start=6ncRNA_end=16
target_start=3372   target_end=3382
ncRNA:       TGTCGGCGCCA
hybrid:      ||||| |||||
target:      GCGGCGGCGGT

>Homo_sapiens_28S_ribosomal_RNA-w4878   Score=138    ncRNA_start=9ncRNA_end=16
target_start=4878   target_end=4885
ncRNA:       TGTCGGCG
hybrid:      ||||||||
target:      GCGGCCGC
```

```
>Homo_sapiens_28S_ribosomal_RNA-w3256   Score=134    ncRNA_start=7ncRNA_end=15
target_start=3256   target_end=3264
ncRNA:        GTCGGCGCC
hybrid:       |||||||||
target:       CAGCTGCGG

>Homo_sapiens_28S_ribosomal_RNA-w2102   Score=123    ncRNA_start=8ncRNA_end=16
target_start=2102   target_end=2111
ncRNA:        TGTCG.GCGC
hybrid:       ||||| ||||
target:       GCGGCGCGCG

>Homo_sapiens_28S_ribosomal_RNA-w3131   Score=123    ncRNA_start=8ncRNA_end=14
target_start=3131   target_end=3137
ncRNA:        TCGGCGC
hybrid:       |||||||
target:       GGCCGCG

>Homo_sapiens_28S_ribosomal_RNA-w241    Score=122    ncRNA_start=8ncRNA_end=16
target_start=241    target_end=250
ncRNA:        TGTCGG.CGC
hybrid:       |||||| |||
target:       GCGGCCGGCG

>Homo_sapiens_28S_ribosomal_RNA-w857    Score=122    ncRNA_start=7ncRNA_end=16
target_start=857    target_end=866
ncRNA:        TGTCGGCGCC
hybrid:       ||||| ||||
target:       GCGGCGGCGG

>Homo_sapiens_28S_ribosomal_RNA-w1865   Score=122    ncRNA_start=7ncRNA_end=16
target_start=1865   target_end=1873
ncRNA:        TGTCGGCGCC
hybrid:       || |||||||
target:       GC.GCTGCGG

>Homo_sapiens_28S_ribosomal_RNA-w2140   Score=122    ncRNA_start=7ncRNA_end=16
target_start=2140   target_end=2149
ncRNA:        TGTCGGCGCC
hybrid:       ||||| ||||
target:       GCGGCGGCGG

>Homo_sapiens_28S_ribosomal_RNA-w3469   Score=122    ncRNA_start=7ncRNA_end=16
target_start=3469   target_end=3478
ncRNA:        TGTCGGCGCC
hybrid:       ||||| ||||
target:       GCGGCGGCGG

>Homo_sapiens_28S_ribosomal_RNA-w4013   Score=122    ncRNA_start=6ncRNA_end=14
target_start=4013   target_end=4022
ncRNA:        TCGGC.GCCA
hybrid:       ||||| ||||
target:       GGCCGCCGGT

>Homo_sapiens_28S_ribosomal_RNA-w488    Score=121    ncRNA_start=7ncRNA_end=16
target_start=488    target_end=496
ncRNA:        TGTCGGCGCC
hybrid:       |||||| |||
target:       GCGGCC.CGG
```
